# The pharmacotherapeutic use of clozapine in older adults: A narrative review

**DOI:** 10.1177/20451253261481517

**Published:** 2026-09-29

**Authors:** Kate Mulholland, Brian O’ Donoghue, Dan Siskind, Geraldine McCarthy, John Lally

**Affiliations:** 1 St Vincent’s Hospital Fairview, Dublin, Ireland; 2 UCD Centre for Psychosis Research, School of Medicine, 8797 University College Dublin, Dublin, Ireland; 3 Department of Psychiatry, St Vincent’s University Hospital, Dublin, Ireland; 4 Addiction and Mental Health Service, Metro South Health, Brisbane, QLD, Australia; 5 Faculty of Health, Medicine and Biomedical Sciences, 1974 University of Queensland, Brisbane, Qld, Australia; 6 Physical and Mental Health Stream, Queensland Centre for Mental Health Research, Brisbane, QLD, Australia; 7 School of Medicine, 8799 University of Galway, Galway, Ireland; 8 Sligo Medical Academy, Sligo University Hospital the Mall, Sligo, Ireland; 9 Department of Psychosis Studies, Institute of Psychiatry, Psychology and Neuroscience (IoPPN), King’s College London, London, UK

**Keywords:** clozapine, treatment resistant schizophrenia, antipsychotic, psychosis, elderly

## Abstract

Clozapine remains the gold-standard treatment for refractory schizophrenia, yet its use in older adults is often limited by concerns regarding tolerability, age-related pharmacokinetic changes, and the burden of medical comorbidities. This narrative review synthesises current evidence on clozapine use in older populations, with a focus on pharmacokinetic alterations, dosing strategies, comorbidity-related considerations, and clozapine’s therapeutic benefits, safety, and risks. A primary aim is to review the nature of age-related pharmacokinetic changes and their cumulative impact over time. Aging is associated with changes in drug absorption, increased volume of distribution due to altered body composition, reduced hepatic blood flow, decreased cytochrome P450 enzyme activity, and impaired clearance, all of which may elevate clozapine and norclozapine plasma concentrations and increase susceptibility to adverse effects. Older adults are particularly vulnerable to sedation, orthostatic hypotension, anticholinergic effects, gastrointestinal hypomotility, falls, and serious adverse events including agranulocytosis and pneumonia. Polypharmacy and comorbidities such as hepatic conditions, obesity and cognitive impairment further complicate treatment and elevate the risk of drug interactions. Evidence supports a conservative “start low, go slow” approach, guided by therapeutic drug monitoring to determine clozapine concentrations, with many older adults achieving therapeutic benefit at lower doses than younger patients, alongside careful monitoring and individualized risk–benefit assessment. Understanding these age-related pharmacokinetic and clinical considerations is essential to optimise dosing, minimise toxicity, and enhance therapeutic outcomes in older patients. Further research is required to strengthen the evidence base and develop clear clinical guidelines for this growing population.

## Plain Language Summary

Clozapine is one of the most effective medications for people with severe schizophrenia that has not improved with other treatments. However, doctors often use it cautiously in older adults because the body changes with age and many older people have other health problems or take several medications. This review explains how aging can affect clozapine treatment. As people get older, the body may process medications more slowly. Changes in liver function, body composition and how the body removes a medication from the system can lead to higher levels of clozapine in the blood, increasing the risk of side effects. Older adults may be more sensitive to side effects such as drowsiness, dizziness when standing up, constipation, falls, confusion, and infections such as pneumonia. Rare but serious side effects, including a reduction in white blood cells (agranulocytosis), can also occur. These risks may be higher in people with other medical conditions, including liver disease, obesity, or memory problems, and in those taking multiple medications. Despite these challenges, clozapine can be a safe and effective treatment for many older adults when used carefully. Doctors usually follow a “start low, go slow” approach, beginning with a low dose and increasing it gradually while monitoring closely for side effects. Regular blood tests and, where appropriate, monitoring the level of clozapine in the blood can help ensure treatment remains safe. Many older adults may achieve good results with lower doses than younger patients. Understanding how aging affects clozapine treatment can help doctors prescribe it more safely and effectively, improving outcomes in older adults with severe mental health conditions like schizophrenia. More research is needed to clearer guidance for the use of clozapine in this growing population.

## Introduction

Schizophrenia in later life is an increasingly important clinical and public health concern. Prevalence estimates in older adults range from 0.1-0.55%,^1–3^ although this likely underestimates the true burden. Approximately 23.5% of schizophrenia cases have onset after the age of 40, classified as late onset schizophrenia, with a smaller proportion with onset after age 60 (very late onset schizophrenia).^4^ Compared with early-onset schizophrenia, very late-onset schizophrenia is characterised by more prominent positive symptoms, fewer negative symptoms, greater delusional conviction and paranoia, and less frequent antecedent affective disturbance.^5–7^

Schizophrenia is associated with a threefold increase in mortality and significantly reduced life expectancy, compared with the general population.^8–11^ Despite this excess premature mortality, the number of older adults living with schizophrenia is expected to rise, with estimates suggesting a doubling of cases aged ≥55 years by 2050^12^ highlighting the need for evidence based guidance on management of schizophrenia in later life.

## Methods

This review was undertaken as an expert consensus narrative review with the aim of synthesising evidence regarding clozapine pharmacokinetics in older adults and developing a practical clinical management framework. Literature was identified iteratively based on clinical relevance, methodological quality, and contemporary applicability, with emphasis placed on large population-based studies, national registry analyses, key observational and interventional studies where relevant, and current international guideline documents. No predefined search period, formal systematic search strategy, or prespecified inclusion and exclusion criteria were applied. This approach is consistent with the objectives of a narrative review and was intended to provide a clinically focused synthesis rather than an exhaustive systematic evaluation of the literature.

### Treatment-resistant schizophrenia in older age

Treatment resistant schizophrenia (TRS) defined as an inadequate clinical response to at least two non-clozapine antipsychotics of adequate dose and duration with confirmed adherence, affects an estimated 30% of individuals with schizophrenia.^13,14^ In first-episode schizophrenia (FES) cohorts, treatment resistance displays two trajectories, primary (or early) resistance, evident from illness onset and affecting approximately 25% of FES patients and secondary (late) resistance, which emerges after an initial response to antipsychotic treatment, affecting 8% of those with schizophrenia.^15,16^ The epidemiology of TRS in older patients remain poorly characterised. Many older patients with TRS represent individuals with illness onset in earlier adulthood years, who have aged into later life, although late-onset TRS also occurs. The clinical complexity of this population is compounded by comorbid physical illness, polypharmacy, and increased vulnerability to adverse drug effects.

### Clozapine as the gold standard for TRS

Clozapine is the gold standard treatment for TRS as reflected in meta-analyses and international clinical guidelines,^13,14,17–19^ yet it is underutilised, with only one-third of eligible patients receiving it in some settings^20^ and with prescribing rates in older adults with schizophrenia not well characterised. Approximately 40% of patients with TRS demonstrate a clinical response to clozapine.^14^ Clozapine’s unique receptor profile, including relatively low D2 receptor affinity and high serotonergic, adrenergic and muscarinic receptor activity confers superior efficacy in TRS and a lower risk of extrapyramidal symptoms, making it particularly suitable for Parkinson’s disease (PD) psychosis and Lewy body dementia, where dopaminergic blockade is poorly tolerated.^21,22^ Clozapine is licensed for PD psychosis^23^ and is used off-label for refractory behavioural and psychological symptoms of dementia (BPSD) under specialist supervision.^24,25^

The use of clozapine in older patients may be limited due to concerns about tolerance and adverse effects. Although evidence in older adults is limited, observational studies and small trials suggest that clozapine remains effective in later life, often at lower doses, with tolerability comparable to younger populations when carefully monitored,^26–34^ and its use in older adults with schizophrenia may be associated with reduced mortality compared to non-clozapine antipsychotics.^35^However, age-related physiological changes substantially alter drug pharmacokinetics, increasing the risk of adverse effects and complicating dosing.

Clozapine has an established role in the management of TRS and PD psychosis across the lifespan, including in older adults. However, the absence of contemporary randomised trials and limited pharmacokinetic data in older age highlight a critical evidence gap. Given the projected growth in the older population with schizophrenia and dementia-related psychosis, further research is urgently needed to inform age-specific dosing strategies, risk-benefit assessment and long-term outcomes. Specifically, no recent reviews on aging effects on clozapine pharmacokinetics is available. This is important as aging related differences in determinants of clozapine pharmacokinetics including CYP enzyme expression, hepatic blood flow, renal excretion and volume of distribution exist and with potential to have impact on clozapine concentrations, response and tolerability (See Table 1).

**Table 1.** Overview of clozapine pharmacodynamic considerations in adults ≥65.

Pharmacodynamic factor	Mechanism in older adults	Clinical implications for clozapine
Altered receptor sensitivity	Age-related increased sensitivity to clozapine’s anticholinergic, α1-adrenergic, serotonergic, histaminergic receptor sensitivity	Increased sensitivity to clozapine’s α1-adrenergic and histaminergic effects, contributing to postural hypotension and sedation; anticholinergic effects (e.g. urinary retention, constipation) are more pronounced
Drug metabolism	Age related changes with slowed metabolism	Prolonged clozapine half-life, action and increased risk of clozapine accumulation
Central nervous system vulnerability	Reduced neuronal reserve and increased physiological vulnerability with ageing	Heightened susceptibility to sedation, cognitive impairment, and falls even at low plasma concentrations; heightened sensitivity to seizures, sedation at increased plasma concentrations
Autonomic dysfunction	Age-related decline in baroreceptor sensitivity and autonomic regulation	Increased risk of orthostatic hypotension, syncope, and falls due to clozapine’s α1-adrenergic blockade
Gastrointestinal motility impairment	Reduced baseline gastrointestinal motility, exacerbated by anticholinergic burden	Increased risk of severe constipation, ileus, and gastrointestinal hypomotility, a major cause of morbidity and mortality with clozapine
Haematological vulnerability	Increased susceptibility to drug-induced cytopenia	Potentially increased risk and severity of neutropenia and agranulocytosis; requires haematological monitoring
Immunological changes	Immunosenescence and higher baseline infection risk in older adults, compounded by clozapine-related sedation, hypersalivation, and aspiration risk	Higher risk of infection (particularly pneumonia), which may be exacerbated by clozapine-induced hypersalivation, sedation and swallowing difficulties
Metabolic sensitivity	Increased prevalence of insulin resistance and metabolic syndrome	Potentially greater risk of clozapine-associated weight gain, dyslipidaemia, and glucose dysregulation
Increased susceptibility to adverse drug effects (ADEs)	Reduced physiological reserve and multimorbidity	Lower threshold for adverse effects; necessitates conservative dosing, slower titration, and close monitoring
Pharmacodynamic interactions	High rates of polypharmacy and comorbid conditions	Increased risk of additive sedation, anticholinergic burden, hypotension, and cardiac effects when combined with other medications

Understanding how these physiological changes affect clozapine pharmacokinetics is therefore essential to inform rational dosing, minimise concentration-dependent toxicity, and optimise therapeutic outcomes in older patients. This review aims to examine the impact of ageing on the pharmacokinetics of clozapine, with particular focus on absorption, distribution, hepatic metabolism, and elimination, and discusses the clinical implications for clozapine prescribing in older adults.

### Clozapine pharmacokinetics in adult (<65) population

Clozapine is a highly lipophilic antipsychotic with complex pharmacokinetics and a narrow therapeutic index.^36,37^ Clozapine pharmacokinetics are clinically important, as plasma concentrations are closely linked to therapeutic efficacy and adverse effects.^38,39^ In advanced age, dose-adjusted clozapine plasma concentrations may be two- to three-fold higher than in middle-aged adults.^40^ Clozapine pharmacokinetics have been more widely studied in younger adults, but evidence specific to older adults remains limited (Table 2).^41–44^

**Table 2.** Clozapine pharmacokinetics in the general population.

Parameter	Value/Observation	Reference
Absorption	Rapid and almost complete after oral administration; tablet and liquid formulations are bioequivalent; minimal impact of food on absorption	^38,45–48^
Bioavailability	Systemic bioavailability: 27–50% due to extensive first-pass metabolism	^48–50^
Time to Maximum Concentration oral tablets (T_max) (mean hours-range)	Clozapine: 3 (range: 1-6 h) N-desmethylclozapine: 9.38 ± 9.33 h (range: 5.00–51.00 h) Clozapine-N-oxide: 4.53 ± 2.19 h (range: 0.00–12.00 h)	^38,48,51^
Typical Clozapine Peak	∼2.5 hours post-dose	^52,53^
Volume of distribution V,L/kg: range	Clozapine 2.5-10 Norclozapine: 9-27	^38,47,48^
Steady-State Achievement	Within ∼5 days following dose adjustment	^38,48,52^
Delayed Gastric Emptying	Occurs in up to 41% of patients; associated with prolonged GI transit and higher serum concentrations	^54,55^
Metabolite Elimination	∼30% eliminated via faeces; severe GI hypomotility may impair biliary elimination and increase systemic exposure	^38,47,48,56^

Clozapine is rapidly and almost completely absorbed following oral administration,^45^ with tablet and liquid formulations demonstrating bioequivalence and minimal food effects.^46^ However, extensive first-pass metabolism limits systemic bioavailability to approximately 27–50%.^49,50^ Peak plasma concentrations typically occur within ∼2.5 hours, although reported time to maximum concentration (T-max) values vary widely across clozapine and its metabolites.^51–53^ Steady state is generally achieved within five days following dose adjustment. Clozapine-induced gastrointestinal hypomotility may delay absorption and prolong T-max, potentially increasing exposure in severe cases, although effects on overall bioavailability remain unclear.^54–56^

Clozapine has extensive tissue distribution, reflected by its large volume of distribution (1.6–7.3 L/kg) and extensive tissue penetration into the central nervous system.^57–59^ It is ∼95% protein bound, primarily to α1-acid glycoprotein,^47,60^ and accumulates in adipose tissue, with higher body weight associated with increased plasma concentrations.^61^

Metabolism is predominantly hepatic via CYP1A2, with contributions from CYP3A4 and CYP2C19, producing the active metabolite norclozapine.^48,62,63^ CYP1A2 activity is the principal determinant of exposure and is influenced by smoking, caffeine, and interacting drugs.^38,64^ Norclozapine has distinct receptor activity, but no established independent antipsychotic effect; its clinical utility is in interpreting the clozapine: norclozapine ratio.^38,65^

Elimination occurs mainly via urine (∼50%) and faeces (∼35%), with minimal unchanged drug excreted.^66^ Clearance declines with age and is lower in women, with substantial inter-individual variability.^67–69^ Clozapine exhibits non-linear pharmacokinetics and prolonged half-life, increasing the risk of accumulation, particularly in older adults.^52^

### Age-related physiological changes affecting clozapine pharmacokinetics

Ageing is associated with progressive physiological changes that alter drug pharmacokinetics and increase interindividual drug concentration variability. Clozapine has a narrow therapeutic index and complex metabolism, making age-related physiological changes particularly relevant for clozapine dosing, therapeutic drug monitoring (TDM), and adverse effect risk in older adults. Age-related changes in absorption, distribution, metabolism, and elimination may contribute to altered drug exposure, increased interindividual variability, prolonged half-life, delayed attainment of steady state, and increased susceptibility to adverse effects in older adults (see Table 3). Understanding these changes is essential to inform rational dosing, optimise TDM, and minimise adverse outcomes in this population.

**Table 3.** Age related pharmacokinetic changes for clozapine and clinical implications.

Pharmacokinetic domain	Parameter	Increased	Decreased	Potential effect on clozapine	Dosing and monitoring recommendations
Absorption	Gastric pH	✓	​	Minimal effect on clozapine solubility and bioavailability	No routine dose adjustment required
	Gastrointestinal blood flow	​	✓	Minimal impact on passive diffusion of clozapine	Not clinically significant
	Gastric acid production	​	✓	Minimal impact on clozapine dissolution	Not clinically significant
	Gastric motility	​	✓	Delayed gastric emptying may prolong time to peak concentration (T_max); increased variability in absorption	Monitor for delayed onset and accumulation in severe hypomotility; consider slower titration
	First-pass metabolism	✓/✓	✓	Reduced first-pass metabolism may increase systemic bioavailability and plasma concentrations	Initiate at low dose; titrate slowly; monitor plasma concentrations
Distribution	Plasma albumin	​	✓	Modest increase in free (unbound) clozapine fraction in frailty or acute illness	Monitor for enhanced pharmacodynamic effects at lower concentrations
	α1-acid glycoprotein	✓	​	Increased protein binding may elevate total (but not free) clozapine concentrations	Interpret total plasma levels cautiously, particularly during inflammation
	Proportion of body fat	✓	​	Increased volume of distribution for lipophilic clozapine; prolonged half-life and accumulation	Lower maintenance doses; slower titration; monitor for delayed toxicity
	Lean body mass	​	✓	Reduced distribution into lean tissue; relative increase in plasma concentrations	Consider lower dose requirements, especially in frail patients
Metabolism	Hepatic blood flow	​	✓	Reduced hepatic clearance leading to increased plasma concentrations	Initiate at low dose; titrate cautiously; use therapeutic drug monitoring (TDM)
	CYP1A2 activity	​	✓	Reduced metabolic clearance; increased clozapine: norclozapine ratio; higher exposure	Avoid rapid dose escalation; adjust dose based on plasma levels and clinical response
	CYP450 interactions	​	✓	Increased susceptibility to drug–drug interactions affecting metabolism	Review concomitant medications; adjust dose when inhibitors/inducers are added or removed
Elimination	Renal function	​	✓	Reduced clearance of clozapine metabolites; potential accumulation	Monitor for increased adverse effects; consider dose reduction in renal impairment
	Glomerular filtration rate	​	✓	Indirect increase in overall drug exposure via metabolite accumulation	Use TDM in patients with declining renal function
	Renal plasma flow	​	✓	Reduced elimination of metabolites	Initiate and maintain lower doses; monitor closely
Additional clinically relevant factors	Inflammation/infection	✓	​	Cytokine-mediated CYP1A2 inhibition leads to rapid increases in plasma clozapine concentrations	Urgent dose reduction and TDM during acute infection
	Smoking status changes	✓/✓	✓	Smoking cessation increases clozapine levels (via CYP1A2 inhibition); smoking induces metabolism	Adjust dose promptly when smoking status changes
	Polypharmacy	✓	​	Additive pharmacokinetic and pharmacodynamic interactions	Regular medication review; increased monitoring frequency
	Frailty and multimorbidity	✓	​	Reduced physiological reserve increases sensitivity to concentration-dependent toxicity	“Start low, go slow”; prioritise tolerability over target dose

#### Absorption

Age-related changes in gastrointestinal (GI) physiology, including delayed gastric emptying and reduced motility, may alter oral drug absorption, though their clinical significance varies by compound. Factors implicated include reduced gastric acidity, delayed gastric emptying, slowed intestinal motility (which may be exacerbated by clozapine), reduced splanchnic blood flow, and changes in GI surface area or luminal content.^70–73^ Reduced gastric acid secretion, previously attributed to ageing, is now thought to reflect factors such as *Helicobacter pylori* infection and widespread proton pump inhibitor use rather than aging, and is unlikely to have a meaningful impact on clozapine absorption.^70^ Splanchnic blood flow has been found to reduce with age,^72^ although these changes do not significantly impact drugs absorbed through passive diffusion such as clozapine.^73^ In practice, clozapine absorption in older adults appears largely preserved but increasingly unpredictable.

Delayed gastric emptying has been observed in older people, particularly in the presence of comorbidities such as diabetes mellitus and renal disease, which impair gastric motility across age groups.^74,75^ Clozapine’s anticholinergic properties, and the frequent co-prescription of other anticholinergic medications, may further exacerbate gastric stasis.^76^ Delayed gastric emptying prolongs the time clozapine tablets are in the stomach for, which may delay T-max. In the presence of gastric stasis or severe gastrointestinal hypomotility, this may increase the risk of erratic drug absorption and systemic accumulation.^38,54,55^

Intestinal motility declines with age and due to clozapine’s own promotion of gastrointestinal hypomotility,^54,55^ older people may be at even greater increased risk of severe constipation, ileus and bowel obstruction.^77,78^ Reduced intestinal motility may lead to delayed small intestine transit, increased contact time with absorptive surfaces, potentially enhancing absorption and increases in bioavailability and plasma clozapine concentrations when gut transit time is prolonged. Conversely, in extreme hypomotility or obstruction, clozapine absorption may become unpredictable or impaired.^49,50,79^

Despite these mechanistic concerns, empirical data on age related differences in gastric emptying and drug absorption between younger and older adults have produced inconsistent findings, with several reporting no significant age-related differences in absorption rate or time to peak concentration.^70,71,73^ Clozapine can be effectively absorbed in older age; however, absorption is characterised by greater variability, with delayed T_max, increased sensitivity to factors that impair GI motility, wider interindividual concentration variability, and increased risk of clozapine toxicity in the presence of severe hypomotility. Despite unchanged bioavailability more variable absorption means closer clinical and plasma clozapine concentration monitoring is required.

#### Distribution

Volume of distribution (V_d) is the ratio of the total amount of a medication in the body to the plasma concentration. Age-related changes in body composition substantially influence V_d. Ageing is associated with reductions in total body water, which predominantly affect hydrophilic medications. In contrast, age related increases in adipose tissue (20-40% increase in fat mass) and reductions in lean body mass (10-15%)^79,80^ have greater relevance for lipophilic drugs such as clozapine, resulting in an increased V_d.^73,81^

Clozapine’s has extensive distribution into adipose tissue, resulting in a large V_d. In older adults, increased adiposity further expands this distribution compartment, resulting in prolonged drug storage in fat tissue and delayed redistribution into the circulation.^57,82^ This has several important pharmacokinetic consequences. Clozapine clearance is proportional to the V_d, and an increased V_d prolongs elimination half-life, delaying both attainment of steady state and drug clearance following dose reduction or discontinuation. It increases the risk of drug accumulation during chronic dosing, particularly in the context of reduced clearance. It may lead to delayed toxicity, as clozapine may be released slowly from adipose tissue following clozapine dose escalation or during intercurrent illness.

Sex-related differences in body composition may further modify these age-related pharmacokinetic changes. Female sex is associated with lower clozapine clearance and higher concentration-to-dose ratios than males, potentially due to differences in CYP1A2 activity, hormonal effects, and body composition.^52,68,83,84^ Population pharmacokinetic studies consistently identify female sex as an independent predictor of higher clozapine concentration-to-dose ratios and reduced clearance, although the relative contributions of body composition and metabolic factors remain incompletely understood.^52,68,83,84^ Postmenopausal changes in adiposity, lean body mass and hormonal status may accentuate these effects, with reductions in oestrogen levels potentially decreasing CYP1A2 activity and alterations in adiposity and lean body mass influencing the volume of distribution (V_d) of clozapine.^85–88^

Protein binding also plays a role in clozapine distribution. Clozapine is approximately 95% protein-bound, primarily to α1-acid glycoprotein (AAG) and, to a lesser extent, albumin. Protein binding also plays a role in clozapine distribution. It has a relatively narrow therapeutic index (compared to other antipsychotic medications) and as such may be susceptible to alterations in protein binding, though this is not well established. Ageing is associated with modest reductions in albumin (10-20%) and increases in AAG related to chronic low-grade inflammation and comorbid medical conditions.^47,59,89^Increased AAG may elevate total plasma clozapine concentrations without increasing the unbound, pharmacologically active fraction,^59,89^ as the rate limiting step in clearance is hepatic metabolism and not protein binding.

Consequently, the clinical relevance of age-related changes in protein binding remains uncertain. While total plasma concentrations may increase, the unbound fraction-the determinant of pharmacological activity-may remain relatively stable under steady-state conditions. However, during acute illness, malnutrition, or hospitalisation, reductions in protein binding capacity may increase the unbound fraction of medications like clozapine, with enhanced pharmacodynamic effects and increased side effects, even at therapeutic or lower clozapine concentrations.^73^ Overall, age-related changes in distribution result in increased V_d, prolonged half-life, and greater interindividual variability in clozapine exposure. These effects are particularly pronounced in older adults with obesity or sarcopenic obesity and have important implications for dosing and monitoring.^57,82^

#### Metabolism and elimination

Ageing is associated with reduced hepatic metabolism due to declines in liver mass, hepatic blood flow, and cytochrome P450 (CYP) enzyme activity. Clozapine undergoes highly variable first pass metabolism.^49,50^ Aging is associated with reduced first pass metabolism, resulting in increased bioavailability. However, no studies have specifically examined how aging impacts clozapine absorption.^90^ Clozapine is primarily metabolised by CYP1A2, with contributions from CYP3A4 and CYP2C19.^38^ Age-related reductions of approximately 20-40% in CYP1A2 activity and 10-25% in CYP3A4 activity have been reported.^91,92^ Decreases in CYP1A2 activity, the primary determinant of clozapine metabolism, with advancing age, results in reduced clearance and higher plasma clozapine concentrations.

Reductions in hepatic volume (20-40%) and hepatic blood flow (40-60%) further decrease hepatic clearance (10-40% for most medications in older adults), contributing to reduced first pass effect, higher peak concentrations (C_max) and prolonged half-life in older adults.^93–95^ Reduced hepatic metabolism results in higher plasma clozapine concentrations at equivalent doses, prolonged elimination half-life, and increased risk of drug accumulation. First-pass metabolism also declines with age, with estimates suggesting a reduction of approximately 1% per year after age 40.^96^ This may contribute to increased systemic exposure following oral administration.

Clozapine exhibits non-linear pharmacokinetics due to saturation of CYP1A2 at higher concentrations; in older adults, reduced enzymatic capacity means saturation may occur earlier or at lower doses, such that relatively small dose increases can precipitate disproportionate increases in plasma concentrations and clozapine: norclozapine ratios in older patients. This non-linearity increases the risk of toxicity during dose escalation and underscores the importance of cautious titration.

Ethnicity may also influence clozapine pharmacokinetics. Asian populations consistently demonstrate higher dose-adjusted clozapine concentrations and lower dose requirements than Caucasian populations, reflecting differences in body composition, smoking prevalence, environmental factors, and CYP1A2 activity.^52,97,98^ Chinese patients were found to require significantly lower clozapine doses than White Caucasian patients (176 mg versus 433 mg/day) to achieve comparable plasma clozapine concentrations,^97^ Similarly, population pharmacokinetic modelling identified Asian ethnicity as an independent predictor of reduced clozapine clearance after adjustment for age, sex, smoking status, and body weight.^52^ However, evidence in older adults remains limited and no age-specific ethnicity-based dosing recommendations currently exist. These findings support careful titration and therapeutic drug monitoring in older Asian patients.

Therapeutic drug monitoring (TDM) studies demonstrate age-related increases in clozapine concentration-to-dose (C:D) ratios. Increasing age is associated with higher clozapine and metabolite concentrations.^99^ A large analysis reported C:D ratio increases of 11% in males aged 50-74 years and 49% in those aged ≥75 years compared with males aged 18-49 years, with corresponding increases of 14% and 28% in females.^83^ The mean clozapine: norclozapine ratio is approximately 1.73,^100^ although this varies widely, it is clinically useful for assessing adherence, dose adequacy and changes in smoking behaviors.^101,102^ The clozapine: norclozapine ratio increases from approximately 1.3 in younger adults to 1.8 in older adults, reflecting reduced metabolic conversion.^103^ These changes are important in relation to raised clozapine concentrations at equivalent does compared to younger adults. Clozapine clearance declines exponentially with age, beginning in the fourth and fifth decade of life.^68^ Population pharmacokinetic modelling further indicates the magnitude of age related changes. Clozapine clearance declines progressively with age from approximately 20.2 L/h at age 20 to 12.0 L/h at age 80.^52^ These changes are attributable to reduced hepatic blood flow, diminished intrinsic CYP1A2 activity, decreased liver mass, and alterations in microsomal function.^104,105^

Although clozapine itself is primarily cleared hepatically, renal function plays an important role in the elimination of its metabolites.^47,69^ Ageing is associated with progressive decline in renal function, characterised by reductions in glomerular filtration rate (GFR), renal blood flow, and tubular secretion. Its metabolites, norclozapine and clozapine N-oxide, undergo renal clearance via tubular secretion and may accumulate in the context of reduced glomerular filtration rate.^47^ While the clinical significance of metabolite accumulation remains unclear, pharmacokinetic modelling suggests increased overall exposure in both hepatic and renal impairment.^106^ Animal models of non-alcoholic fatty liver disease demonstrate reduced norclozapine formation and increased metabolite exposure without significant changes in parent drug levels, highlighting the potential contribution of comorbid hepatic disease in older adults.^107^ Increases in clozapine metabolites may contribute to overall adverse effect burden, although this relationship remains poorly characterised, particularly in older patients where data are limited.^108^ Although the clinical significance of metabolite accumulation remains uncertain, reduced renal function may compound the effects of reduced hepatic clearance and contribute to overall drug exposure.

### Effect of comorbid diseases on clozapine pharmacokinetics in older adults

Older adults with schizophrenia frequently have multiple comorbid conditions that further influence clozapine pharmacokinetics. Obesity increases the volume of distribution (V_d) and is associated with higher plasma clozapine concentrations. Hepatic impairment reduces metabolic capacity, while renal dysfunction may impair metabolite clearance. These factors often coexist and contribute to increased variability and toxicity risk.

Obesity significantly alters clozapine pharmacokinetics, with relevance in older adults who exhibit higher rates of increased body mass index (BMI), metabolic syndrome, and adiposity.^109,110^ Clozapine’s lipophilicity results in extensive distribution into adipose tissue, leading to increased V_d and reduced clearance.^57,90^ Observational data demonstrate that higher BMI is associated with increased plasma clozapine concentrations, with reported increases of 18–21% in individuals with higher BMI,^82^ with weight and body fat independently associated with increased clozapine concentrations.^57,84^ Although there are no pharmacokinetic studies examining clozapine in sarcopenic populations, pharmacokinetic evidence from other drugs suggests that sarcopenia is associated with reduced V_d and increased peak plasma concentrations (Cmax), with no consistent effect on time to peak concentration (Tmax)^111^ and higher risk of toxicity.^112^ These findings indicate that sarcopenia meaningfully influences drug distribution.

Frailty is associated with reduced physiological reserve and may alter drug distribution, metabolism, and clearance independently of chronological age.^113–116^ These changes can increase pharmacokinetic variability and susceptibility to adverse effects.^115^ Frail older adults may therefore be particularly vulnerable to clozapine accumulation and treatment intolerance, supporting cautious dosing, slower titration, and closer monitoring.

Hepatic metabolism is central to clozapine clearance, and age-related liver dysfunction further increases exposure risk. Preclinical models suggest clozapine may exacerbate fatty liver disease and metabolic dysfunction,^117^ while clinical reports describe reversible hepatitis and rare hepatic failure^118,119^ Routine liver function monitoring is therefore essential, particularly in older adults and those with pre-existing hepatic impairment.^120,121^

Renal impairment, common in ageing, may also increase clozapine exposure. While the parent drug is hepatically metabolised, its metabolites are renally cleared and may accumulate.^47^ Although norclozapine and clozapine-N-oxide do not have established antipsychotic efficacy, accumulation of these metabolites may contribute to adverse effects and may serve as markers of altered clozapine disposition. Reduced renal function may therefore contribute indirectly to overall toxicity risk, particularly when combined with reduced hepatic clearance. Modelling studies predict increased bioavailability in renal dysfunction, supporting lower dosing, slow titration, and TDM.^106,122^

Additional variability may arise from menopause-related hormonal changes affecting CYP1A2 activity,^123^ though clinical findings remain inconsistent. Declining oestrogen levels during the peri- and postmenopausal period may increase clozapine metabolism, potentially reducing plasma clozapine concentrations.^124^ Despite these mechanistic considerations, empirical data examining menopause-specific effects on clozapine pharmacokinetics remain limited and inconsistent. A large retrospective study found a 60% decline in clozapine concentrations in women aged 40-60 years, with no comparable change in men.^85^ Other population-based analyses have shown that clozapine exhibits the greatest age-related increase in serum concentrations among antipsychotics, with women having 20-30% higher levels than men in later life.^40^ These findings indicate that older, postmenopausal women are particularly vulnerable to variations in and to elevated clozapine exposure, yet no consensus dosing guidance currently exists for this population.

Drug interactions-particularly CYP1A2 inhibitors (e.g. fluvoxamine) and inducers (e.g. rifampicin)-substantially influence clozapine levels, necessitating careful monitoring.^125,126^ SSRIs with minimal cytochrome inhibition, such as escitalopram, are therefore preferred in older patients receiving clozapine.^127,128^ (See supplementary table 1).

Infections, more common and severe in older adults, can markedly elevate clozapine levels via cytokine-mediated CYP suppression, increasing toxicity risk. Dose adjustment and close monitoring during acute illness are therefore critical.^64,129^ Infection represents a critical and often under-recognised determinant of clozapine toxicity, particularly in older adults who are more susceptible to both infection and its complications due to age-related immune decline.^130^ Clozapine is independently associated with an increased risk of infections, particularly pneumonia, due to increased sedation, hypersalivation, and swallowing difficulties with increased risk of aspiration and aspiration pneumonia.^129,131,132^ Epidemiological data highlight the scale of this risk, with pneumonia a leading cause of death among clozapine-treated patients.^77,132^

Importantly, acute infection can significantly elevate clozapine plasma concentrations through cytokine-mediated suppression of CYP1A2 activity and increased production of acute-phase proteins such as α1-acid glycoprotein.^133,134^ These changes reduce metabolic clearance and alter protein binding, leading to rapid increases in clozapine concentrations. Case reports describe substantial rises in clozapine levels during infections, including up to fourfold increases.^89,135^ These changes markedly increase the risk of concentration dependent adverse effects such as sedation, hypersalivation, seizures, delirium and cardiovascular complications. Accordingly, proactive dose reduction, close clinical observation, and TDM are essential during infection or when initiating antibiotics to mitigate toxicity and maintain treatment continuity.^126,129^

## Pharmacokinetic changes with aging and clozapine concentrations

Age-related pharmacokinetic changes necessitate a fundamentally different approach to clozapine dosing in older adults compared with younger populations. Reduced hepatic clearance, increased V_d, and altered protein binding contribute to higher plasma concentrations at lower doses, prolonged half-life, and increased inter-individual variability. These factors collectively mandate conservative dosing strategies, slow titration, and routine TDM.

Multiple clinical and pharmacokinetic modelling studies confirm that ageing is independently associated with reduced clozapine clearance, resulting in higher dose-adjusted plasma concentrations.^40,52,84,99,103,136,137^ Reeves et al. conducted the largest population pharmacokinetic analysis to date, examining 17,787 plasma clozapine and norclozapine samples from 5,960 patients aged 18-86 years across the UK and Ireland.^52^ Using a model incorporating age, smoking status, body weight, sex, and ethnicity, it demonstrated that clozapine clearance declines significantly with age, resulting in higher plasma concentrations at equivalent doses.^52^ Importantly, the dose required to achieve therapeutic plasma concentrations decreased by approximately 56% between the ages of 20 to 80 years, with a median prescribed dose of 200-300 mg/day in patients over 65, and even lower dose requirements in those over 85 (See Table 4).^52^ Dose correction factors of 0.60-0.70 were recommended for individuals aged 80 years relative to younger adults.^52^

**Table 4.** Clozapine dose adjustments by age, sex and smoking status.^52^

Age (years)	Sex	Smoking Status	Predicted Dose (mg/day)	Notes
40	Male	Non-smoker	275	Reference adult
40	Male	Smoker	358	+30% smoking
40	Female	Non-smoker	228	−17% female
40	Female	Smoker	296	−17% female + 30% smoker
80	Male	Non-smoker	120	Predicted dose decreased by 56% between age 20 -80 years
80	Male	Smoker	156	+30% smoking
80	Female	Non-smoker	100	−17% female
80	Female	Smoker	129	−17% female + 30% smoking

Consistent with these findings, Bowskill et al. examined TDM data from patients aged ≥65 years and reported wide interindividual variability in plasma clozapine concentrations relative to prescribed dose, with over one-third of plasma clozapine concentrations above the therapeutic concentration range despite lower prescribed doses.^103^ Notably, median daily doses decreased with advancing age (65-70 years: median dose 300mg/day vs ≥85 years: median dose 200mg/day), yet plasma concentrations remained high, with 37% of samples exceeding the therapeutic range (0.35-0.60 mg/L) reflecting reduced clozapine clearance.^103^

Similarly, earlier work by Rostami-Hodjegan et al. using a predictive model based on 3,782 plasma samples, demonstrated that plasma clozapine concentrations increase by approximately 4% for every five-year increment in age above 40 years, independent of other factors.^84^ Similar findings have been reported in large observational datasets, with dose-adjusted concentrations significantly increased in older age groups.^40,138^ A large retrospective TDM study of 17,249 samples reported dose-adjusted plasma clozapine concentrations increased by 108% at age 80 and 197% at age 90 compared to those at age 40, with females exhibiting concentrations 20-30% higher than males.^40^

Large observational studies confirm that serum concentration-to-dose (C:D) ratios increase significantly with age.^138^ In a retrospective cohort of 3734 clozapine treated patients including 1089 patients aged 45-59 years and 111 aged 60-75 years, the older patients received lower daily clozapine doses yet exhibited significantly higher plasma clozapine concentrations compared with younger adults (10–44 years), resulting in a marked age-related increase in C:D ratios (p<0.001).^138^ Ismail et al. reported declining clearance of both clozapine and norclozapine beyond the age of 39 years (n=391 patients aged 11-79 years (2,284 samples).^68^ In contrast, Li et al. found no age-related pharmacokinetic effects in a younger Chinese cohort (aged 18-59 years), suggesting that age effects may emerge predominantly in later life.^139^

These findings reflect age-associated physiological changes, particularly reduced hepatic clearance and increased V_d, which collectively predispose to drug accumulation and heightened toxicity risk if doses are not appropriately adjusted.^33,140,141^

Pharmacokinetic consequences of ageing include prolongation of clozapine half-life to approximately 20-36 hours and norclozapine half-life to 40-69 hours, with time to steady state extending from 5 days in younger adults^53^ to 7-10 days in older individuals.^142^

### Clinical considerations in older adults

#### Effectiveness of clozapine

Clozapine can be effective in older adults with TRS^143^ with evidence largely derived from observational and cohort studies. Prospective and retrospective studies suggest that a substantial proportion of older patients experience meaningful symptom improvement and are able to sustain treatment over time.^144,145^ Additionally, similar discontinuation rates between older and younger adults in cohort analyses may indicate comparable overall effectiveness when clozapine is appropriately managed.^140^ However, the evidence base remains predominantly observational, often involving small and clinically heterogeneous samples and tolerability remains a key clinical challenge.

#### Tolerability of clozapine

Retrospective cohort studies provide broader insight into tolerability. A cohort study of 284 patients with TRS found that older adults (>55 years) reported fewer adverse effects during the first three months of treatment compared with younger patients (18-55 years), although extrapyramidal symptoms were reported more often in the older group.^140^ Importantly, rates of discontinuation due to adverse effects were similar between age groups (23.0% vs 21.8%),^140^ suggesting that clozapine may not be inherently less tolerable in older adults when appropriately managed.

Recent studies report adverse effects in up to 94% of older clozapine-treated patients, with constipation among the most frequent complications.^34^ Seven patients discontinued clozapine due to planned discontinuation as part of end of life care (n=2) and side-effect burden (n=5). Nine deaths occurred while taking clozapine, none of which were attributed to medication.^34^ An earlier prospective cohort study in 329 patients aged ≥55 years (mean age 63.4, SD=6.5 years; range 55 to 86 years) identified a discontinuation rate of 43% (n=107) due to administrative issues (n=32), poor response (n=19), patient noncompliance (n=17) central nervous system side effects (n=12), haematological side effects (n=7) and cardiovascular side-effects (n=6). Five deaths occurred, two of unclear etiology and three due to underlying medical illness.^144^

However, other studies highlight the high burden of adverse effects in this population. In a retrospective review of patients aged 65-89 years (n=75), clozapine was discontinued in 49% of cases, most commonly due to death (unrelated to clozapine) (n=14), non-fatal adverse events (n=12), patient choice (n=8), or unspecified reasons (n=3).^145^ A recent retrospective study in clozapine treated patients over 65 (n=69) reported that 94% of patients experienced at least one adverse effect in the preceding year.^34^

#### Adverse effects

Common but clinically significant adverse effects such as sedation, hypersalivation, constipation and gastrointestinal hypomotility, urinary incontinence, and orthostatic hypotension are more pronounced in older patients and may lead to functional decline or treatment discontinuation.^146^ Sedation is common and may be more pronounced in older patients, contributing to falls, functional decline and aspiration risk through antihistaminergic and anticholinergic mechanisms.^146^ Older adults are also more susceptible to orthostatic hypotension due to α1-adrenergic blockade and age-related autonomic dysfunction, increasing the risk of syncope, falls and fractures, particularly during treatment initiation or dose escalation.^147^

Older adults may be particularly vulnerable to the metabolic consequences of clozapine because of age-related increases in adiposity, insulin resistance and reduced metabolic reserve. These factors may amplify the clinical impact of clozapine-associated weight gain and dyslipidaemia.^80,110,141^ Gastrointestinal hypomotility is a major concern, ranging from constipation to life-threatening ileus.^54,55^ Older adults may be particularly vulnerable, with constipation reported in up to 65% of patients in one recent cohort.^34^ Hypersalivation may further compound aspiration risk, particularly when combined with sedation.^64,77,126,132^ Metabolic complications and dose-related seizures are also important concerns and may reflect higher plasma exposure in later life.^148^

Age-related physiological vulnerability, polypharmacy and comorbidities contribute to an increased burden of adverse effects, many of which are dose- and concentration-dependent. Importantly, the adverse effect profile in older adults differs qualitatively and quantitatively from that observed in younger populations, with greater susceptibility to anticholinergic, cardiovascular, metabolic, and haematological complications,^141,146,149^ although overall discontinuation rates may be similar.^140^ Serious complications, including agranulocytosis, seizures, and myocarditis carry higher morbidity and mortality risks.^64,129,132,150^ Risks such as myocarditis and agranulocytosis are most clinically relevant during early stages of clozapine treatment, as their incidence is highest in the early phases of treatment, including in older adults; in contrast the risk is significantly lower for those entering later life established on maintenance clozapine. Although the absolute risk of agranulocytosis is low, it may increase with age.^151^

Agranulocytosis occurs in approximately 1% of clozapine-treated patients,^152^ and increased rates of clozapine-induced agranulocytosis have been observed in older patients. Earlier estimates suggested that the risk may increase by 53% for every 10-year increase in age at clozapine initiation.^151^ The higher incidence of neutropenia in older adults likely reflects the cumulative impact of multiple risk factors, including greater exposure to concurrent medications associated with bone marrow suppression, age-related reductions in marrow reserve and comorbid conditions that impair granulopoiesis or increase peripheral granulocyte destruction. This interpretation is consistent with evidence demonstrating a substantially increased risk of idiosyncratic drug-induced neutropenia in individuals aged over 65 years.^153,154^

#### Management of adverse effects

Management of clozapine-related adverse effects in older adults should not rely solely on dose reduction, as excessive dose reduction may compromise symptom control and increase the risk of relapse in TRS. Instead, treatment should aim for the lowest effective dose while balancing efficacy and tolerability. Slower titration, smaller dose increments, and regular assessment of blood pressure, sedation, bowel function, cognition, and falls risk may improve tolerability and facilitate early recognition of adverse effects. Dividing the daily dose, where clinically appropriate, may help reduce peak-related adverse effects such as excessive sedation and orthostatic hypotension.

Medication review should focus on agents that increase sedation, orthostatic hypotension, anticholinergic burden, constipation risk, or alter clozapine exposure. Caution is warranted with benzodiazepines, opioids, anticholinergic medications, and other sedative agents. Regular deprescribing of non-essential medications should be considered to minimise cumulative adverse-effect burden. (Supplementary Table 1).

### Dosing and therapeutic drug monitoring (TDM)

Age-related pharmacokinetic changes necessitate dosing strategies in older adults that prioritise safety and personalised dosing. Clozapine should be initiated at low doses, typically 6.25mg once daily given at night, with slower titration than in younger adults and with smaller dose increments and longer intervals between adjustments. A practical approach is to initiate clozapine at 6.25-12.5 mg at night and increase by 6.25-12.5 mg increments every 2-3 days, according to tolerability, blood pressure, sedation, and other adverse effects. Slower titration may be required in frail individuals, those with cardiovascular disease, or those receiving multiple interacting medications.

Given the high prevalence of sedation and orthostatic hypotension with clozapine, particularly in older adults, the majority of the daily dose is often administered at night to reduce daytime sedation and minimise the impact of postural hypotension on daytime functioning.^126,146,155^ Dose splitting may improve tolerability in patients with persistent sedation, dizziness, or orthostatic symptoms. Dosing should be individualised according to clinical response, comorbidities, and adverse effect burden.

Typical maintenance doses in older adults range from 125mg to 300 mg/day, although higher doses may be required in some cases^29,145,156^ and lower doses typically used in PD psychosis,^157–160^ with vigilant monitoring of plasma clozapine concentrations to attain therapeutic concentrations. Some patients may require higher doses to achieve therapeutic response, particularly those with longstanding illness who were initiated on clozapine earlier in life. However, in older adults, doses above 350 mg/day are associated with an increased risk of supratherapeutic plasma concentrations and adverse effects.^52,103^ Dose requirements decrease progressively with age, with reductions of more than 50% observed between early adulthood and advanced age.^52^ This may mean that for clozapine maintenance treatment, as a patient ages, consideration for dose reductions to maintain stable clozapine concentrations will be needed.

Given the delayed attainment of steady state in older adults, plasma clozapine concentrations should generally be measured after at least 5-7 days on a stable dose, and repeated following significant dose changes, intercurrent illness, smoking changes, or the introduction of interacting medications. Initial clozapine concentration monitoring may be considered once patients have reached a stable dose (often within the range of 50-100 mg/day).

A suggested clozapine titration schedule for individuals older than 65 with TRS is provided in box 1.

**Box 1. Suggested Clozapine Titration in Adults ≥65 years**

**Table:** 

Time period	Dose (mg/day)	Dosing schedule
Day 1	6.25-12.5 mg	Once daily at night
Day 2–3	6.25-12.5 mg	Maintain if tolerability concerns; otherwise increase cautiously
Day 4–7	12.5-25 mg	Once daily or divided doses depending on tolerability
Week 2	25-50 mg	Increase by 6.25-12.5 mg every 2-3 days as tolerated
Week 3	50-75 mg	Divided doses if required; monitor sedation, orthostatic hypotension, and other adverse effects
Week 4+	75-100 mg and onward (individualised)	Consider initial plasma clozapine concentration monitoring once a stable dose has been reached, often within the 50–100 mg/day range. Measure concentrations after at least 5–7 days on a stable dose and repeat after significant dose changes, intercurrent illness, changes in smoking, or introduction of interacting medications
Maintenance (older adults)	Typically 50-200 mg/day	Use the lowest effective dose; lower doses may be required in frail older adults or Parkinson’s disease psychosis. Some patients may require higher doses (200-300mg/day)

Higher doses may be prescribed for patients who were commenced on clozapine in adulthood and remained on the dosage into old age.^142^ Currently, there are no formal guidelines to direct clinicians on whether to maintain or reduce clozapine doses in older adults. It is advised to commence clozapine at a lower dose in adults aged over 65 years old than in younger adults, and to slowly titrate the dose in line with haematological monitoring, with a suggested target dose of 50-200mg.^149,156^ Ongoing dose reduction may be required as patients age to maintain stable plasma concentrations. Antipsychotic polypharmacy should generally be avoided in older adults receiving clozapine, as evidence supporting efficacy is limited while risks of adverse effects, drug interactions, falls, cognitive impairment, and cumulative anticholinergic burden are increased.^161,162^

TDM is central to clozapine management in older adults. Plasma clozapine concentrations provide an objective measure of drug exposure and are closely associated with both clinical response and toxicity. Target therapeutic ranges (typically 0.35–0.60 mg/L^38,39^) should be interpreted in the context of individual patient factors, including age, comorbidities, and clinical response. The clozapine: norclozapine ratio provides additional clinical information, particularly in assessing adherence, metabolic capacity, and drug interactions. Increased ratios in older adults reflect reduced metabolic conversion and may indicate increased risk of clozapine accumulation.^38^

Given the increased pharmacokinetic variability in older adults, TDM is essential following dose adjustments, the introduction of interacting medications, or the onset of medical comorbidities, in particular intercurrent illness or infections. Comprehensive assessment of comorbidity burden should precede clozapine initiation in older adults. Notably, the absence of large randomised controlled trials in this population means current prescribing guidance is largely informed by pharmacokinetic data and observational evidence. Accordingly, conservative dosing strategies-characterised by lower starting doses, slower titration, and close monitoring of plasma concentrations-are recommended to optimise safety and efficacy in later life.

## Conclusion

Clozapine can be safely prescribed in older patients when age related pharmacokinetic and pharmacodynamic changes are carefully considered. Increased V_d, impaired hepatic metabolism and reduced clearance all contribute to higher clozapine concentrations and prolonged half-life. Age related heightened sensitivity to adverse effects may limit tolerability and further enhance need for personalised clozapine dosing in older patients with regular TDM.

These biological aging changes highlight the need for careful clozapine dosing and dose adjustments, informed by TDM to ascertain clozapine levels, to reduce the risk of concentration dependent adverse effects. Clozapine prescribing in older adults should be individualised rather than guided by chronological age alone. Frailty, comorbidities, organ function, concomitant medications, therapeutic response, adverse effects, and patient preferences should all inform treatment decisions. While pharmacokinetic principles and TDM provide valuable guidance, clinical judgement remains central to achieving the optimal balance between efficacy and safety. Individualised dosing strategies and TDM are essential to minimise toxicity while preserving efficacy, underscoring the need for formalised clinical guidelines for clozapine use in the older population.

## Supplemental Material

Supplemental Material - The pharmacotherapeutic use of clozapine in older adults: A narrative review Supplemental Material for The pharmacotherapeutic use of clozapine in older adults: A narrative review by Kate Mulholland, Brian O’ Donoghue, Dan Siskind, Geraldine McCarthy, John Lally in Therapeutic Advances in Psychopharmacology.

## Data Availability

Not applicable. This manuscript is a narrative review and does not involve the generation or analysis of original datasets. All information presented is derived from published literature, which is referenced within the manuscript.
