# Supplementary material for: The pharmacotherapeutic use of clozapine in older adults: A narrative review
Source: Ther Adv Psychopharmacol. 2026 Sep 29;16:20451253261481517. doi: 10.1177/20451253261481517 (PMC13769913; doi:10.1177/20451253261481517)
Supplement: Supplementary file 1 [file sj-pdf-1-tpp-10.1177_20451253261481517.pdf]

Supplementary Table 1: Medications Affecting Clozapine Plasma Levels

Medication/Class	Effect on Clozapine Levels	Mechanism	Clinical Implications / Notes	References
**Fluvoxamine (SSRI)**	↑ Clozapine; ↓ Norclozapine; ↑ Clozapine: norclozapine ratio	CYP1A2 inhibition	Dose adjustments recommended; small doses (<25 mg/day) preferred; effect magnitude variable	[126,164]
**Fluoxetine, Paroxetine (SSRIs)**	↑ Clozapine increased risk of toxicity	CYP1A2 inhibition (less than fluvoxamine);	Substantial increases in plasma concentrations, increased toxicity risk	[128,129,165–169]
**Escitalopram, Sertraline**	Minimal effect	Minimal CYP inhibition	Preferred SSRIs in older patients on clozapine	[128,129]
**Carbamazepine (Anticonvulsant)**	↓ Clozapine (~47%)	CYP1A2 & CYP3A4 induction	Reduced plasma levels, risk of relapse; haematological toxicity risk	[170,171]
**Phenytoin, Phenobarbital**	↓ Clozapine (~35%)	CYP induction	Dose adjustment/ TDM essential	[172–174]
**Ciprofloxacin (Fluoroquinolone)**	↑ Clozapine	CYP1A2 inhibition	Rapid, potentially fatal increases	[175–177]
**Erythromycin (Macrolide)**	↑ Clozapine	CYP1A2 inhibition	Significant rise in levels; monitor for toxicity	[178]
**Rifampicin (Antibiotic)**	↓ Clozapine	CYP1A2 & CYP3A4 induction	Reduced levels → risk of relapse; dose adjustment required	[179,180]
